# Integrated photonics enables continuous-beam electron phase modulation

**DOI:** 10.1038/s41586-021-04197-5

**Published:** 2021-12-22

**Authors:** Jan-Wilke Henke, Arslan Sajid Raja, Armin Feist, Guanhao Huang, Germaine Arend, Yujia Yang, F. Jasmin Kappert, Rui Ning Wang, Marcel Möller, Jiahe Pan, Junqiu Liu, Ofer Kfir, Claus Ropers, Tobias J. Kippenberg

**Affiliations:** 1 Georg-August-Universität Göttingen, Göttingen, Germany; 2 Max Planck Institute of Multidisciplinary Sciences, Göttingen, Germany; 3 Swiss Federal Institute of Technology Lausanne (EPFL), Lausanne, Switzerland; 4 Center for Quantum Science and Engineering, EPFL, Lausanne, Switzerland

**Keywords:** Quantum metrology, Quantum optics, Sub-wavelength optics, Matter waves and particle beams

## Abstract

Integrated photonics facilitates extensive control over fundamental light–matter interactions in manifold quantum systems including atoms^1^, trapped ions^2,3^, quantum dots^4^ and defect centres^5^. Ultrafast electron microscopy has recently made free-electron beams the subject of laser-based quantum manipulation and characterization^6–11^, enabling the observation of free-electron quantum walks^12–14^, attosecond electron pulses^10,15–17^ and holographic electromagnetic imaging^18^. Chip-based photonics^19,20^ promises unique applications in nanoscale quantum control and sensing but remains to be realized in electron microscopy. Here we merge integrated photonics with electron microscopy, demonstrating coherent phase modulation of a continuous electron beam using a silicon nitride microresonator. The high-finesse (*Q*_0_ ≈ 10^6^) cavity enhancement and a waveguide designed for phase matching lead to efficient electron–light scattering at extremely low, continuous-wave optical powers. Specifically, we fully deplete the initial electron state at a cavity-coupled power of only 5.35 microwatts and generate >500 electron energy sidebands for several milliwatts. Moreover, we probe unidirectional intracavity fields with microelectronvolt resolution in electron-energy-gain spectroscopy^21^. The fibre-coupled photonic structures feature single-optical-mode electron–light interaction with full control over the input and output light. This approach establishes a versatile and highly efficient framework for enhanced electron beam control in the context of laser phase plates^22^, beam modulators and continuous-wave attosecond pulse trains^23^, resonantly enhanced spectroscopy^24–26^ and dielectric laser acceleration^19,20,27^. Our work introduces a universal platform for exploring free-electron quantum optics^28–31^, with potential future developments in strong coupling, local quantum probing and electron–photon entanglement.

## Abstract (web summary)

A silicon nitride microresonator is used for coherent phase modulation of a transmission electron microscope beam, with future applications in combining high-resolution microscopy with spectroscopy, holography and metrology.

## Main

The rapid advancement of electron microscopy epitomizes our growing ability to characterize the structure and function of nanoscale materials, devices and biological systems. Beyond stationary imaging, the advent of in situ and time-resolved electron microscopy allows for the observation of transient phenomena and non-equilibrium dynamics^32–35^. In the form of photon-induced near-field electron microscopy (PINEM)^6^, ultrafast transmission electron microscopy (UTEM) permits quantitative, high-resolution imaging of nano-optical fields^7,36–38^. The underlying mechanism involves energy transfer between localized optical excitations and free electrons, modifying the state of the electron by generating discrete photon sidebands in a process termed stimulated inelastic electron-light scattering (IELS). The corresponding longitudinal phase modulation of the electronic wavefunction at optical frequencies is quantum-coherent in nature^8,12^, and thus can be used for the coherent control of electron quantum states in space^9,11,39,40^ and time^10,16,17^. Recent theoretical works suggest that such modulated electron beams enable an electron-mediated transfer of optical coherence, predicting the generation of coherent cathodoluminescence, the resonant excitation of two-level systems and superradiance from sequential electrons^24,25,41–43^. These developments create opportunities for a closer integration of electron microscopy with coherent optical spectroscopy, based on local quantum control and enhanced sensing. Harnessing coherent electron–light interactions for scientific and technological applications is, however, hampered by its usual limitation to the ultrafast regime. Recent works implemented IELS^44,45^, a ponderomotive laser phase plate^22^ and an attosecond modulator^23^ at high continuous-wave powers. Low coupling efficiencies and structural damage have thus far limited inelastic scattering to weak interactions. Despite the use of phase matching and resonant amplification in dielectric laser accelerators^19^, prism geometries^46,47^ or free-space coupled whispering-gallery-mode microresonators^48^, achieving strong phase modulation of an electron beam has remained out of reach of regular electron microscopes.

**Fig. 1:**
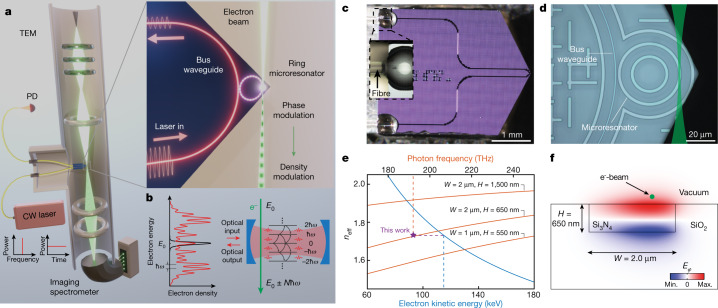
Principle of continuous-wave photonic-chip-based optical phase modulation of free-electron beams. **a**, Rendering of the experimental set-up, including the electron microscope and a fibre-coupled Si_3_N_4_ photonic-chip-based microresonator. Inset: magnified interaction region with the electron beam passing the microresonator. CW, continuous wave; PD, photodiode. **b**, Left: during interaction with the laser-driven cavity mode, the initially narrow electron spectrum (black) develops discrete sidebands at integer multiples of the photon energy (red). Right: in a cQED depiction, the cavity photons induce transitions between the free-electron energy ladder states. **c**, Photograph of the fibre-coupled Si_3_N_4_ photonic chip mounted on a customized TEM holder. The triangular-shaped chip edge minimizes undesired electron–substrate interactions (the inset image shows the optical fibre glued to the input waveguide). **d**, Optical microscope image of the photonic chip showing the bus waveguide and the microresonator. The electron beam (green path, not to scale) traverses the microresonator parallel to the chip surface. **e**, Frequency-dependent effective index (*n*_eff_) of the fundamental quasi-TM microresonator mode (orange). The integrated on-chip platform allows for phase matching to be achieved at different electron kinetic energies (blue, 90–145 keV) either by changing the dimensions of the Si_3_N_4_ waveguide or operating at different optical frequencies. For the waveguide shown in **f**, phase matching is achieved between the optical mode at ~193.5 THz (dashed orange line, corresponding to a wavelength of ~1,550 nm) and the free electrons at ~115 keV (dashed blue line). *W*, width; *H*, height. **f**, FEM simulation of the *E*_*φ*_ distribution of the fundamental quasi-TM mode of the microresonator (the green dot is an exemplary electron trajectory pointing into the page).

Here, we overcome this challenge and demonstrate highly efficient electron–photon interactions in the continuous-wave regime using an electron microscope and photonic integrated circuits based on Si_3_N_4_. Our set-up (Fig. 1) allows an electron beam to interact with the copropagating evanescent field of a microresonator waveguide in the object plane of a transmission electron microscope (TEM).

## Free-electron–cavity photon interaction

The electron–photon interaction at the photonic chip (schematic in Fig. 1) is described by the Hamiltonian^28,29^ (see derivation in the Supplementary Information)

1 $${H}_{{\rm{int}}}=\frac{e}{2m}(\hat{{\bf{p}}}\cdot \hat{{\bf{A}}}+\hat{{\bf{A}}}\cdot \hat{{\bf{p}}})=\hbar {g}_{0}\hat{{\bf{a}}}{\hat{{\bf{b}}}}^{\dagger }+\hbar {g}_{0}^{\ast }{\hat{{\bf{a}}}}^{\dagger }\hat{{\bf{b}}}.$$

While the length gauge is usually chosen for localized quantum systems in cavity quantum electrodynamics (cQED)^49^, the above velocity gauge is a natural choice for free electrons at finite momentum $\hat{{\bf{p}}}$ in an electromagnetic field with vector potential $\hat{{\bf{A}}}$. The interaction between the optical mode ($\hat{{\bf{a}}},{\hat{{\bf{a}}}}^{\dagger }$: annihilation/creation operator) and an electron ($\hat{{\bf{b}}},{\hat{{\bf{b}}}}^{\dagger }$: electron-energy ladder operators, see Supplementary Information) is characterized by the vacuum coupling rate *g*_0_ (refs. ^28,29^), determined by the mode distribution and the matching of electron group and optical phase velocities:

2 $${g}_{0}=\eta \sqrt{\frac{{e}^{2}{v}_{{\rm{e}}}^{2}}{2\varepsilon \hbar \omega V}}.$$

Here, *e* is the electron charge, *v*_e_ the electron group velocity, *ε* the optical permittivity, *ω* the angular frequency and *V*  the effective optical mode volume. The phase matching condition is manifested in the coefficient $\eta =\int {\rm{d}}z{{\rm{e}}}^{-i\Delta k\cdot z}u(z)/L$   defined by the optical mode profile function *u*(*z*) describing the electric field component along the electron trajectory, linked to the vector potential by $\hat{{\bf{A}}}=\hat{{\bf{A}}}(z)=\sqrt{\frac{\hbar }{2\varepsilon \omega V}}(u(z)a+{u}^{\ast }(z){a}^{\dagger }),$ and the electron wavenumber change Δ*k* = *ω*/*v*_e_ upon photon absorption and emission^8,50^. The scattering matrix, summarizing the effect of the interaction on both the electron and the cavity, is given by $S={\rm{\exp }}(-i{g}_{0}\tau \hat{a}{\hat{b}}^{\dagger }-i{g}_{0}^{\ast }\tau {\hat{a}}^{\dagger }\hat{b}\,)$^28^. An empty cavity, therefore, facilitates electron-driven photon generation^28,51,52^ in a spontaneous Cherenkov or Smith–Purcell-like process^53^, with coherence properties characteristic of transition radiation and so-called coherent cathodoluminescence^54^. Populated by a coherent state $|\alpha \rangle$ from a laser, the free-electron–photon interaction reduces to a dimensionless coupling parameter *g* = *iταg*_0_, where |*α*|^2^ = *n*_*c*_ is the mean intracavity photon number, *τ* = *L*/*v*_e_ is the electron’s transit time over the interaction region *L,* and the scattering operator becomes a displacement operator acting on the electron state^12^. The interaction produces electron energy sidebands separated from the initial energy *E*_0_ by integer multiples of the photon energy $N\hbar \omega$ (*N* ∈ ℤ, where *N* is the photon order), with populations *P*_N_ following the Bessel functions of the first kind, *P*_*N*_ = *J*_*N*_(2|*g*|)^2^ (refs. ^12,50^). In a position representation, the interaction imprints a sinusoidal phase modulation onto the electron wavefunction^50^, which upon dispersive propagation will result in a density modulation of the electron beam^10,16,17^.

## Chip-based integrated photonics platform

To facilitate high interaction strengths, we use photonic chip-based Si_3_N_4_ microresonators, a platform with many important features, including radiation hardness, high power handling^55^, extremely low propagation losses (below 1 dB m^−1^ at 1,550 nm)^56^ and flexibility, to engineer dispersion for phase matching. The chip was fabricated using the photonic Damascene process^56,57^ (Methods) without top oxide cladding to allow for efficient free-electron–light interactions with the evanescent field. The ring microresonator of 20 μm radius is coupled to a bus waveguide and placed close to a triangular edge of the chip to minimize undesired electron–substrate interactions (Fig. 1d and Methods). For operation in the TEM, the photonic structure was packaged via ultrahigh numerical aperture (UHNA) fibres (Fig. 1c and Methods). The Si_3_N_4_ microresonators employed here enable phase matching at different electron energies by modifying the waveguide geometry (Fig. 1e); various established integrated platforms could further extend the phase matching range in terms of the electron energy and optical frequency (see Supplementary Information).

In the current study, we designed a ring microresonator (cross-section: 2 μm × 650 nm) to provide phase matching at an optical frequency of ~193 THz (wavelength *λ* ≈ 1,549 nm) for a target electron energy of 115 keV. Figure 1f shows a finite-element method (FEM, Methods) simulation of the quasi-transverse magnetic (quasi-TM) mode profile in terms of its major contributing field component *E*_*φ*_ along the electron propagation direction. Owing to the small mode area and considerable evanescent field component, we predict a vacuum coupling rate of *g*_0_/2π ≈ 10^11^ Hz over an interaction time of *τ* ≈ 10^−13^ s (*L* ≈ 19 μm). The microresonator’s high *Q* factor facilitates a unity coupling constant *g* ≈ 1 (the coupling constant *g* is a complex number; for simplicity, we use *g* in place of $|g|$ hereafter) at a coupled optical power of *P* = *n*_*c*_*ħωκ* ≈ 1 μW, where *κ*/2π = 390 MHz is the measured cavity decay rate inside the electron microscope (the intrinsic *Q* factor of *Q*_0_ ≈ 0.74 × 10^6^ was slightly degraded in the microscope, see Methods).

## Combined optical and electron spectroscopy

**Fig. 2:**
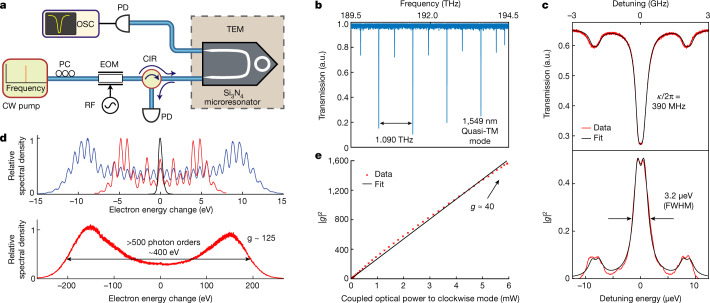
Simultaneous optical and electron spectroscopy of a high-*Q* microresonator mode. **a**, A continuous-wave laser was used to excite the quasi-TM mode of the Si_3_N_4_ microresonator by using a polarization controller (PC). The relative optical frequency was calibrated by imparting sidebands (±2 GHz) via an electro-optic phase modulator (EOM). The total transmitted and back-reflected light was detected to calibrate the power coupled into the clockwise propagating mode. CIR, circulator; OSC, oscilloscope; RF, radio-frequency synthesizer. **b**, Normalized transmission scan of the microresonator quasi-TM mode measured outside the TEM with a *Q* factor of ~0.77 × 10^6^ (*κ*_0_/2π = 112 MHz, external coupling loss rate *κ*_ex_/2π = 139 MHz) and a free spectral range of ~1.090 THz (see Methods for in situ optical characterization). **c**, Simultaneously measured optical transmission at the output waveguide (top) and |*g*|^2^ retrieved from the electron energy spectra (bottom) while the electron beam interacted with the evanescent cavity field. The measured |*g*|^2^ trace follows the power coupled to the clockwise mode; a slight splitting is present due to modal coupling. FWHM, full-width at half-maximum. **d**, Example electron energy spectra for low (top; *g* = 0 (black), *g* ≈ 3.5 (red) and *g* ≈ 6.7 (blue)) and high (bottom, *g* ≈ 125) optical powers. **e**, |*g*|^2^ varies linearly with the optical power coupled to the clockwise mode of the cavity (slope: |*g*|^2^ = *P*/3.70 μW). a.u., arbitrary units.

**Fig. 3:**
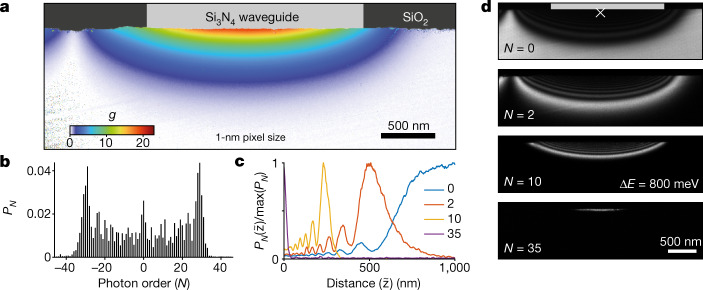
High-resolution hyperspectral imaging of the quasi-TM microresonator mode. **a**, Spatial map of *g* at phase matching (115 keV electron energy) with a 1 nm image resolution. **b**, Exemplary electron spectrum retrieved from the energy-filtered spatial map (position indicated by a cross in **d**). **c**, *N*-dependent sideband population as a function of the distance $\tilde{z}$ to the chip surface. **d**, Energy-filtered images of selected *N* (indicated). The energy window has a width of 800 meV and the position of the microresonator is indicated in grey.

**Fig. 4:**
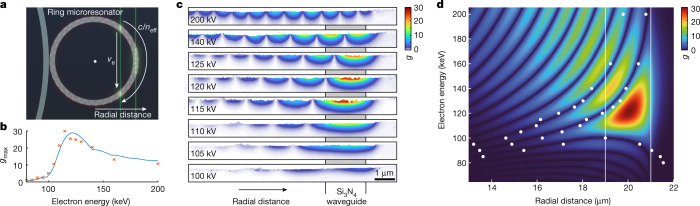
Phase matching and Ramsey-type interference. **a**, Geometry (side view) of the electron beam passing the photonic chip and interacting with the quasi-TM microresonator mode. The double interaction and phase shifts between the light and electron wave led to a characteristic spatial distribution of *g* due to Ramsey-type interference. **b**, Comparison of experimental (red crosses, see **c**) and numerically simulated (blue line, see **d**) maximum *g* as a function of the electron kinetic energy. **c**, Quantitative *g* maps for variable electron energies, showing the spatial distribution and amplitude of the electron–light interaction. The radial position of the Si_3_N_4_ waveguide is indicated. **d**, Numerical simulation of *g* as a function of electron energy and lateral position just above the surface. The white dots overlay the experimentally observed minima retrieved from **c** (80–200 kV electron beam energies, the waveguide positions are marked by white lines).

In the experiments, the fibre-coupled microresonator is driven by a 1,550 nm continuous-wave laser via the bus waveguide. It is mounted on a customized sample holder and placed in the object plane of the field-emission TEM (Fig. 1a and Methods). Parallel to the surface, the electron beam passes the waveguide and interacts with the confined optical mode (inset in Fig. 1a). After traversing the structure, the electron kinetic energy distribution is characterized with an imaging electron spectrometer in two different ways. Specifically, high-dispersion electron energy spectra are recorded by positioning a sharply focused electron beam in front of the microresonator. Alternatively, energy-filtered TEM using a collimated beam is used to image the interaction across the entire cavity mode in the near field. Whereas the former yields electron spectra for varying experimental parameters (Fig. 2), the latter enables imaging of individual sideband populations with high spatial resolution (Figs. 3 and 4).

We first investigate the strength of *g* using a focused electron probe, recording electron spectra while scanning the laser over a resonance employing the set-up depicted in Fig. 2a. The transmission spectrum, displayed in Fig. 2b, shows the quasi-TM microresonator modes spaced by a free spectral range of 1.090 THz. Figure 2c shows simultaneous optical and in situ electron spectroscopy of the laser-excited mode. An electro-optical modulator (driven at 2.0 GHz) is used to generate sidebands that can be observed in the transmission spectrum to calibrate the optical frequency. The laser is tuned to a single optical mode at ~1,549.4 nm (*κ*/2π = 390 MHz, Methods) and the focused electron beam (120 keV beam energy, 25 nm focal spot size, 1 mrad convergence semi-angle) is centred just above the surface of the microresonator (Fig. 3) to record electron spectra for a stationary beam. Harnessing the high-*Q* intracavity enhancement, we observe strong populations *P*_*N*_ in multiple *N*, reaching the regime of strong phase modulation for a continuous laser light source and electron beam (Fig. 2d). These results relate to sideband generation in a recent experiment using semi-transparent membranes^23^ and a study employing dielectric gratings posted at the same time as our work ^14^. The coupling parameter *g* is retrieved from the spectra (Methods), whereas the optical power coupled to the clockwise-propagating mode is determined from the recorded optical transmission and reflection data (Fig. 2e). We observe the expected linear dependence |*g*|^2^ = *P*/3.70 μW of the coupling on the in-coupled clockwise optical power. From this, we find that an optical power of 5.35 μW is required to suppress the electron zero-energy-loss peak at *g ≈* 1.2, and a value of *g ≈* 40 for an optical power of about 6 mW. For a power of ~38 mW in the bus waveguide, we generate >500 photon sidebands (*g ≈* 125, Fig. 2d). While state-of-the-art dielectric laser accelerators achieve the highest peak acceleration gradients for sub-relativistic electron beams using femtosecond lasers^19,58^, our approach enables continuous acceleration of microbunched electron beams^10^,^26^ with a gradient of 330 keV m^–1^ per milliwatt of optical power. In terms of the input peak power required, this is an improvement in efficiency of four orders of magnitude over free-space coupled dielectric structures^47,48^.

Representing a particular strength of the approach introduced here, integrated photonics permits the controlled interaction with a single optical mode, as described in the following. The spectral line shape of the resonance is analysed by simultaneously recording the transmitted optical power and extracting *g* for varying frequency detuning (Fig. 2c). The optical transmission trace, formed by the interference of the input light with the light coupled out from the clockwise-propagating resonator mode, exhibits a full-width at half-maximum of 560 MHz (total line width *κ*/2π = 390 MHz, Supplementary Information). Interestingly, the electron spectra, which are sensitive only to the intracavity power stored in the clockwise-rotating, copropagating optical mode, display a double-peaked structure originating from coupling the power to the frequency-degenerate counterclockwise mode^59^. The differences in width and shape between the optical and electron spectroscopic measurements are explained by the interference in the optical transmission channel with the input field, and both curves can be fitted consistently in one model as presented in the Supplementary Information (Supplementary Figs. 1 and 2). These data demonstrate continuous-wave electron energy gain spectroscopy^21,60^ at an extraordinarily narrow spectral feature of only 3.2 μeV in width (full-width at half-maximum; 1 μeV peak separation).

## Mode imaging and phase matching

The implementation of chip-based high-*Q* optical microresonators as efficient electron phase modulators requires insights into the real-space scattering distribution. Employing a collimated electron beam and an imaging energy filter (800 meV energy pass band), we record spatial maps of *P*_*N*_ (Fig. 3d, Methods). A photon-order resolved spectrum is extracted for each image pixel (Fig. 3b), and *g* is retrieved with a 1 nm spatial resolution (Fig. 3a). Using a continuous electron beam circumvents previously encountered signal-to-noise ratio limitations in PINEM at about 3–4 orders of magnitude lower flux. The distance-dependent sideband occupations (Fig. 3c) reveal a strong and high-contrast modulation, indicating the absence of spatial and temporal averaging. We note that IELS is typically accompanied by a three-dimensional momentum transfer^9,11^; however, in the current experiment, these transverse deflections did not limit the resolution for in-focus imaging (Supplementary Information).

The microresonator waveguide was designed to match the phase velocity of the excited optical mode with the group velocity of electrons at an energy of around 115 keV. Figure 4 shows the spatial pattern of the electron–light coupling near the ring-shaped microresonator (Fig. 4a). The electron-energy-dependent near-field maps reveal an oscillatory modulation of *g* along the chip surface (Fig. 4c) and an amplitude change with electron energy (Fig. 4b). At phase matching, the spatial distribution of *g* closely resembles the electric field configuration of the excited quasi-TM mode (Fig. 3a), and the most efficient coupling is achieved for a beam passing the periphery of the ring (tangent line). Trajectories closer to the centre of the microresonator (secant line) are governed by two sequential interactions with the microresonator mode. This results in Ramsey-type constructive or destructive interference that depends on the relative phase of both individual interactions^13^.

Numerical FEM simulations of the optical mode profile (Fig. 4d, Methods), considering the electron trajectory through the near field and the microresonator power enhancement, closely reproduce the experimental observations, including the position of the minima in *g* (white dots). Excellent agreement is achieved for the electron energy dependence of the maximum *g* (Fig. 4b). In contrast to nanoscale-confined optical fields, a steep cutoff in electron–light coupling is found for beam energies below ~100 keV because the optical mode is devoid of higher-momentum electric field components. At high electron energies, the coupling strength reaches a plateau, which enables broadband temporal phase plates for TEM. Besides enhancing the total coupling via a larger interaction time, an increase in microresonator radius will further sharpen the phase matching condition in energy.

## Conclusion

In summary, we demonstrate highly efficient, phase-matched and single-optical-mode interaction of free electrons with photonic-chip-based high-*Q* microresonators, driving transitions at microwatt continuous optical pump powers. Simultaneous in situ optical and electron measurements show that this cQED-type setting yields a quantitative understanding of the interaction and facilitates electron energy gain spectroscopy at the microelectronvolt level. Our approach may enable a seamless integration of temporal phase plates in electron microscopy over a broad range of energies, with various applications in advanced longitudinal and transverse electron beam shaping for high-fidelity attosecond metrology and sub-cycle probing, beam blanking and high-frequency beam modulation. Coherent beam separation in the optical field may enable new variants of inelastic electron holography, and sequential interactions will provide sensitivity enhancements in coherent electron spectroscopy. We envisage various possible architectures featuring anomalous dispersion to directly probe the multimode intracavity field in nonlinear effects such as the formation and dynamics of dissipative Kerr solitons, or coupled microresonators for advanced electron state preparation and readout. The present study provides a missing link in the realization of various theoretical concepts in free-electron cQED by establishing full control over the optical input and output channels of a single confined integrated microresonator mode coupled to an electron beam. Future investigations will address cavity-mediated electron–photon and electron–electron entanglement using coincidence detection, promising enhanced electron imaging and electron-heralded single-photon sources.

## Methods

### Numerical simulations

The effective refractive index (*n*_eff_) and the mode profile of the Si_3_N_4_ microresonator are calculated via FEM simulations (COMSOL Multiphysics) (Fig. 1f). Mode analysis is performed for the two-dimensional axially symmetric model with the cross-section of the microresonator. The Si_3_N_4_ core has a rectangular cross-section with width *W* and height *H*. The microresonator has a SiO_2_ bottom cladding and an air (vacuum) top cladding. The refractive index of Si_3_N_4_ used in the simulation was obtained from in-house ellipsometry measurements and found to be *n*_*K*_ = 1.9923 (J. A. Woollam, unpublished data), whereas the refractive index of SiO_2_ was obtained from ref. ^61^ as *n*_*M*_ = 1.4440. From the electric field distribution in the cross-section ($r,\tilde{z}$), the complex electric field along the electron trajectory ${E}_{{\rm{traj}}}(r,\tilde{z},z)\,,$ related to the physical field by *E*_physical_ = real[*E*_traj_], is determined via  ${E}_{{\rm{traj}}}(r,\tilde{z},z)={E}_{\phi }(r,\tilde{z})\cos (z/r)+{E}_{r}(r,\tilde{z})\sin (z/r)$. The coupling strength $g(r,\tilde{z},E)$ is then found by evaluating $g=\frac{e}{2\hbar \omega }{\int }_{-\infty }^{\infty }{E}_{{\rm{traj}}}(r,\tilde{z},z)\exp \left(-i\frac{\omega }{{v}_{{\rm{e}}}}z\right){\rm{d}}z$ (refs. ^12,50^) for different electron energies *E*, where ${v}_{{\rm{e}}}=c\sqrt{1-1/{(1+E/{m}_{0}{c}^{2})}^{2}}$ is the relativistic electron velocity.

### Fibre-integrated silicon nitride microresonators

The microresonators used in this study are made from Si_3_N_4_, a platform that is CMOS compatible with radiation hardness capability^62^ and ultra-low propagation losses^56,57^. They also allow for dispersion engineering and efficient fibre-to-chip coupling. Si_3_N_4_-based integrated photonic circuits have been driving major progress in nonlinear optical devices, in particular soliton microcombs^63^ that are already used in numerous system-level applications ranging from coherent telecommunication^64^ to astrophysical spectrometer calibration^65,66^. Here, the designed microresonators were fabricated using the photonic Damascene process^56,57^. A 4-inch silicon substrate with 4-μm-thick thermal wet SiO_2_ cladding was used. The substrate was coated with deep-ultraviolet (DUV) photoresist, and microresonators and bus waveguides were patterned on the substrate via DUV stepper photolithography (248 nm KrF excimer laser). The pattern was then dry-etched into the SiO_2_ cladding using C_4_H_8_/O_2_/He etchants to create waveguide preforms. Stoichiometric Si_3_N_4_ film was deposited on the patterned substrate via low-pressure chemical vapour deposition (LPCVD), to fill the waveguide preforms and to form the waveguide cores. Afterwards, etchback and chemical-mechanical polishing^56^ were used to planarize the substrate and remove excess Si_3_N_4_. The entire substrate was further annealed at 1,200 °C to remove the residual hydrogen content in Si_3_N_4_, to reduce hydrogen-induced absorption loss in the Si_3_N_4_ waveguides. This high-temperature annealing is critical to fabricate low-propagation-loss waveguides at telecommunication bands around 1,550 nm. No top SiO_2_ cladding was added on top of the Si_3_N_4_ waveguides, such that the electron beam could interact with the optical mode. Finally, photolithography with alignment was used to precisely position the Si_3_N_4_ microresonator close to the chip edge (within a distance <10 μm, key for aligning the electron beam with the Si_3_N_4_ microresonator). Deep reactive-ion etching was used to separate the entire substrate into hundreds of individual dies/chips for the following die-level integration with fibres.

The light is coupled into and out of the Si_3_N_4_ photonic chip on one edge, while the microresonator is placed on the other edge. To avoid cutting the electron beam by the substrate (Fig. 1c), the near edge was clipped, forming a tight supporting triangle around the resonator ring to avoid damage during the chip release. A bus waveguide (dimensions 800 nm × 650 nm) is used to evanescently couple the light into the multimode microresonator (dimensions 2 μm × 650 nm) to achieve a better coupling ideality^67^. Inverse-taper waveguides^68^ are used to facilitate efficient light coupling via UHNA-7 fibres (mode field diameter ≈ 3.2 μm at 1,550 nm). A 2–3-cm-long UHNA-7 fibre with a thermally expandable core was spliced to standard single-mode fibre (SMF-28) with a splicing loss of <0.2 dB. A chip through-coupling efficiency (fibre–chip–fibre) of >25% is achieved using the UHNA-7 fibre. The photonic packaging was done by first aligning the UHNA-7 fibre via a custom-built holder to optimize the coupling. Then, a small drop of epoxy (~150 μm, Fig. 1b) was dispensed using a precise pneumatic valve and cured using a UV lamp in four small time steps (~2–5 min). A long-term (~1–2 days) coupling stability test was performed at low optical power by monitoring the transmitted light. The broadband characterization of the microresonators was performed by employing a widely tunable diode laser. The transmission spectrum was calibrated using a self-referenced optical frequency comb and Mach–Zehnder interferometer (MZI)^69^. The resonance is fitted using models explained in the Supplementary Information (fitting model) to extract *κ*_0_/2π (intrinsic loss rate), *κ*_e*x*_/2π (external coupling loss rate), and *γ*/2π (mode coupling rate) for both quasi-TE and quasi-TM mode families. The mean intrinsic linewidth is ~110–120 MHz (*Q*_0_ ≈ 1.75 × 10^6^) for the quasi-TM mode family. The cavity resonance centre at ~1,549 nm is critically coupled with *κ*_0_/2π = 112 MHz and *κ*_ex_/2π = 139 MHz (*Q* ≈ 0.77 × 10^6^).

### Optical characterization of microresonators inside the TEM

The packaged sample is transferred into the TEM using a customized holder with vacuum fibre feedthroughs. The fibres connected to the microresonator chip are fed through the hollowed pipe part of the holder, and the T-shaped base holding the photonic chip is mounted on an adaptor that allows the placement of the entire structure in the sample region of the TEM. The optical set-up used to perform the spectroscopy in the TEM is shown in Fig. 2a. The set-up is driven by a CW-laser (Toptica CTL 1550), with a maximal power of 40 mW, a tunable wavelength from 1,510 nm to 1,630 nm, and a linewidth below 10 kHz. The laser is coupled to an electro-optic modulator (EOM) that allows for frequency calibration of the transmission scan and the dependence of *g* on the detuning from the resonance frequency. A polarization controller is used to align the input light to either the quasi- TE or TM mode of the microresonator. An optical circulator is used to probe the light reflected from the microresonator due to the bulk and Rayleigh surface scattering responsible for the splitting of clockwise and counterclockwise cavity modes. The optical transmission and reflection are measured simultaneously to calibrate the intracavity power coupled to the clockwise mode (see Supplementary Information). We note that a degradation of the *Q* factor from 0.77 × 10^6^ to 0.49 × 10^6^, resulting in an additional increase in total linewidth (~150 MHz), was observed when the sample was transferred into the TEM, which could be related to some charging, strain or contamination of the resonator chip. Theory predicts that |*g*|^2^ is proportional to the photon number *n*_cw_ of the clockwise mode optical cavity mode. The intracavity photon numbers are varied by sweeping the laser frequency through the optical resonance to find the |*g*|^2^ dependence on optical power. The *g*(*t*) value is extracted for each time step of the sweep, *t*, by fitting the measured electron spectrum, and the intracavity photon number *n*_cw_(*t*) is retrieved from the optical transmission signal using the steady-state Langevin equation. By binning the |*g*(*t*)|^2^ trace in 20 ms steps and plotting it against the coupled optical power in the clockwise mode $P\,=\,\kappa \hbar \omega {n}_{{\rm{cw}}}(t)$, we obtain the power sweep curve shown in Fig. 2e. More detailed data analysis procedures and the analytical expression of various field quantities can be found in the Supplementary Information.

### Electron microscope and experimental set-up

The in situ experiments were performed in the Göttingen UTEM^70^, which is based on a thermal field-emission TEM (JEM 2100F, JEOL Ltd). Achieving temporal resolutions in the femtosecond regime^70–72^, UTEM has enabled stroboscopic real-space movies of numerous processes, including strain wave dynamics^34,73,74^, ultrafast demagnetization^75–77^ and the evolution of structural phase transformations^33,35^. In the current experiments, the electron gun is operated in the extended Schottky regime, yielding a continuous electron beam with an initial energy spread of 0.5 eV at variable electron energies from 80 to 200 keV and typical beam currents of 10–50 pA in the sample region. Preventing clipping of the electron beam at the extended microresonator chip (~50 μm sample height at the probing position), a low beam convergence is set by turning off the magnetic objective lens in low magnification (LM) TEM and STEM mode, for high-resolution imaging and spectroscopy, respectively. For analysing the electron beam passing the microresonator structure, a post-column imaging energy filter (CEFID, CEOS) is employed, equipped with a scintillator-coupled CMOS camera (TemCam-XF416ES, TVIPS GmbH; used for all measurements, if not mentioned otherwise) and a hybrid-pixel electron detector (EM CheeTah T3, Amsterdam Scientific Instruments). The sample tilt is adjusted carefully to align the electron beam parallel to the surface of the microresonator chip, probing the local electron–light interaction at the microresonator.

### Local spectroscopy of the microresonator mode

Electron spectroscopy (Fig. 2) is implemented in LowMAG STEM mode (indicated magnification: ×1,000) at 120 keV electron energy, achieving an electron focal spot size of about 25 nm (FWHM) with beam semi-convergence angles of 1.1 mrad (100 μm condenser aperture, Fig. 2d) and 0.45 mrad (40 μm condenser aperture, Fig. 2c (lower panel) and e), respectively. The electron beam is either positioned about 30 nm over the microresonator surface, such that no clipping or beam distortion was observed (Fig. 2d), or at a distance of 380 nm from the microresonator surface (Fig. 2c, e), as estimated from the optical power in the bus waveguide and the exponential decay of the optical mode. We observe some charging of the dielectric microresonator chip when using a tightly focused electron beam. Therefore, larger distances to the surface or fast beam sweeps are employed, and future structures will address this point by adding a thin conductive coating on parts of the microresonator chip. The STEM focal plane is set to the middle of the ring microresonator and the lateral beam position relative to the chip is software controlled (Filter Control, CEOS) via an external scan generator (USG, TVIPS). Single spectra are recorded with an integration time of 100 ms (Fig. 2d). Electron spectroscopy with fast sweeps of the laser frequency is captured by an event-based hybrid pixel detector (EM CheeTah T3, Timepix3 ASIC, 1.56 ns timing precision) and discrete binning in time (100 μs windows, Fig. 2c, e). For each spectrum, the electron–light coupling constant *g* is extracted by fitting the shape and amplitudes of the individual spectral sidebands *N* with a comb of Voigt-peaks and normalized occupations *P*_*N*_ = *J*_*N*_(2|*g*|)^2^. For details on the fitting procedure, see refs. ^12,48^. By considering the change of the coupling constant *g* as a function of frequency detuning, the optical linewidth can be extracted, and the achievable energy resolution in electron energy-gain spectroscopy is ultimately limited by the laser linewidth. The concept can be transferred to arbitrary high-*Q* optical modes, with previously demonstrated spectral linewidths below *κ*/2π ≈ 10 MHz (ref. ^56^). In comparison, state-of-the-art monochromated EELS probes material excitations with a zero-loss peak (ZLP) of sub-10-meV energy width^78–80^.

### Energy-filtered imaging and quantitative *g* maps

Quantitative maps of the electron–light coupling constant *g* are retrieved by energy-filtered transmission electron microscopy (EFTEM) imaging (Figs. 3 and 4), resolving individual photon sidebands with a double-sided energy window of only 800 meV width and typical bi-sided r.m.s. non-isochromaticity (energy window edge sharpness) of about 30 meV (8 mm field of view of the spectrometer entrance aperture plane, for technical details, see ref. ^81^). Changing the microscope’s high tension (step size of 0.8 V) relative to the centre beam energy (80–200 keV) allows for the recording of images *I*_*N*_(*x*, *y*) that contain inelastically scattered electrons, losing or gaining an integer number *N* of photon energies *ħω* = 0.8 eV (Fig. 3c). For each spectral sideband, an image is recorded with 1-s integration time, resulting in energy-filtered TEM datasets containing up to 121 image slices, enabling a quantitative retrieval of the coupling constant *g* at each image pixel (see Fig. 3d). Accounting for a large number of image positions (up to 4,096 × 4,096 pixels in Fig. 3), the data are collocated into 1,024 subsets using the *k*-means++ clusters algorithm (implemented in MATLAB 2020b, MathWorks Inc.), with the sideband occupations as the input vector. The resulting centroid locations (metric: squared Euclidean distances) give a set of averaged spectra. For each of these, the coupling constant *g*, relative uncertainty *dg*/*g* (with standard deviation *dg* of Gaussian distribution in *g*) and spectral amplitudes *A* are fitted to the recorded image stacks *I*_*N*_(*x*, *y*). The averaged intensity spread of the ZLP to neighbouring energy windows, *B*, is accounted for by applying a convolution *I*_*N*_(*x*, *y*) = *A*(*x*, *y*) ⋅ *P*_*N*_(*x*, *y*) * *B* (at 115 keV beam energy, the 800 meV energy window contained about 82% of the ZLP intensity). Furthermore, remaining ZLP intensity at stronger coupling (*g* > 3), resulting from limited rejection contrast of the spectrometer slits and scattering at the entrance aperture, is disregarded (about 2(0.8)% diffuse background for *N* = 0( ± 1)). Finally, the retrieved fitting parameters are assigned to the individual pixels before clustering, resulting in a quantitative map of the electron–light coupling constant with 1 nm spatial resolution (Figs. 3 and 4).

## Online content

Any methods, additional references, Nature Research reporting summaries, source data, extended data, supplementary information, acknowledgements, peer review information; details of author contributions and competing interests; and statements of data and code availability are available at 10.1038/s41586-021-04197-5.

## Supplementary information

Supplementary Information This Supplementary Information file contains Sections 1–4, including Supplementary Figures 1–3 and additional references. Section 1: Analytical equations used for fitting optical resonance; Section 2: Quantum optical description of electron–photon interaction; Section 3: Estimation of transverse beam detection; Section 4: Integrated photonics platforms for electron beam modulation.

## Data Availability

The data that support the plots within this paper and other findings of this study are available on Zenodo (10.5281/zenodo.5575752). All other data used in this study are available from the corresponding authors upon reasonable request.

## References

[1] Thompson JD (2013). Coupling a single trapped atom to a nanoscale optical cavity. Science.

[2] Mehta KK (2020). Integrated optical multi-ion quantum logic. Nature.

[3] Niffenegger RJ (2020). Integrated multi-wavelength control of an ion qubit. Nature.

[4] Lodahl P, Mahmoodian S, Stobbe S (2015). Interfacing single photons and single quantum dots with photonic nanostructures. Rev. Mod. Phys..

[5] Sipahigil A (2016). An integrated diamond nanophotonics platform for quantum-optical networks. Science.

[6] Barwick B, Flannigan DJ, Zewail AH (2009). Photon-induced near-field electron microscopy. Nature.

[7] Piazza L (2015). Simultaneous observation of the quantization and the interference pattern of a plasmonic near-field. Nat. Commun..

[8] García de Abajo FJ (2010). Optical excitations in electron microscopy. Rev. Mod. Phys..

[9] Vanacore GM (2018). Attosecond coherent control of free-electron wave functions using semi-infinite light fields. Nat. Commun..

[10] Priebe KE (2017). Attosecond electron pulse trains and quantum state reconstruction in ultrafast transmission electron microscopy. Nat. Photon..

[11] Feist A, Yalunin SV, Schäfer S, Ropers C (2020). High-purity free-electron momentum states prepared by three-dimensional optical phase modulation. Phys. Rev. Res..

[12] Feist A (2015). Quantum coherent optical phase modulation in an ultrafast transmission electron microscope. Nature.

[13] Echternkamp KE, Feist A, Schäfer S, Ropers C (2016). Ramsey-type phase control of free-electron beams. Nat. Phys..

[14] Dahan R (2021). Imprinting the quantum statistics of photons on free electrons. Science.

[15] Sears CMS (2008). Production and characterization of attosecond electron bunch trains. Phys. Rev. Spec. Top. Accel. Beams.

[16] Morimoto Y, Baum P (2018). Diffraction and microscopy with attosecond electron pulse trains. Nat. Phys..

[17] Kozák M, Schönenberger N, Hommelhoff P (2018). Ponderomotive generation and detection of attosecond free-electron pulse trains. Phys. Rev. Lett..

[18] Madan I (2019). Holographic imaging of electromagnetic fields via electron-light quantum interference. Sci. Adv..

[19] England RJ (2014). Dielectric laser accelerators. Rev. Mod. Phys..

[20] Sapra NV (2020). On-chip integrated laser-driven particle accelerator. Science.

[21] García de Abajo FJ, Kociak M (2008). Electron energy-gain spectroscopy. New J. Phys..

[22] Schwartz O (2019). Laser phase plate for transmission electron microscopy. Nat. Methods.

[23] Ryabov A, Thurner JW, Nabben D, Tsarev MV, Baum P (2020). Attosecond metrology in a continuous-beam transmission electron microscope. Sci. Adv..

[24] Gover A, Yariv A (2020). Free-electron–bound-electron resonant interaction. Phys. Rev. Lett..

[25] Zhao Z, Sun X-Q, Fan S (2021). Quantum entanglement and modulation enhancement of free-electron–bound-electron interaction. Phys. Rev. Lett..

[26] Yalunin SV, Feist A, Ropers C (2021). Tailored high-contrast attosecond electron pulses for coherent excitation and scattering. Phys. Rev. Res..

[27] Shiloh R (2021). Electron phase-space control in photonic chip-based particle acceleration. Nature.

[28] Kfir O (2019). Entanglements of electrons and cavity photons in the strong-coupling regime. Phys. Rev. Lett..

[29] Di Giulio V, Kociak M, de Abajo FJG (2019). Probing quantum optical excitations with fast electrons. Optica.

[30] Pan Y, Gover A (2019). Spontaneous and stimulated emissions of a preformed quantum free-electron wave function. Phys. Rev. A.

[31] Ben Hayun A (2021). Shaping quantum photonic states using free electrons. Sci. Adv..

[32] Zewail AH (2010). Four-dimensional electron microscopy. Science.

[33] van der Veen RM, Kwon O-H, Tissot A, Hauser A, Zewail AH (2013). Single-nanoparticle phase transitions visualized by four-dimensional electron microscopy. Nat. Chem..

[34] Cremons DR, Plemmons DA, Flannigan DJ (2016). Femtosecond electron imaging of defect-modulated phonon dynamics. Nat. Commun..

[35] Danz T, Domröse T, Ropers C (2021). Ultrafast nanoimaging of the order parameter in a structural phase transition. Science.

[36] Yurtsever A, van der Veen RM, Zewail AH (2012). Subparticle ultrafast spectrum imaging in 4D electron microscopy. Science.

[37] Wang K (2020). Coherent interaction between free electrons and a photonic cavity. Nature.

[38] Liebtrau M (2021). Spontaneous and stimulated electron– photon interactions in nanoscale plasmonic near fields. Light Sci. Appl..

[39] Vanacore GM (2019). Ultrafast generation and control of an electron vortex beam via chiral plasmonic near fields. Nat. Mater..

[40] García de Abajo FJ, Konečná A (2021). Optical modulation of electron beams in free space. Phys. Rev. Lett..

[41] Gover A (2019). Superradiant and stimulated-superradiant emission of bunched electron beams. Rev. Mod. Phys..

[42] Di Giulio, V., Kfir, O., Ropers, C. & García de Abajo, F. J. Modulation of cathodoluminescence emission by interference with external light. *ACS Nano* acsnano.1c00549 (2021). 10.1021/acsnano.1c00549 PMC8939848 33724007

[43] Kfir O, Di Giulio V, de Abajo FJG, Ropers C (2021). Optical coherence transfer mediated by free electrons. Sci. Adv..

[44] Das P (2019). Stimulated electron energy loss and gain in an electron microscope without a pulsed electron gun. Ultramicroscopy.

[45] Liu C (2019). Continuous wave resonant photon stimulated electron energy-gain and electron energy-loss spectroscopy of individual plasmonic nanoparticles. ACS Photon..

[46] Kozák M (2017). Acceleration of sub-relativistic electrons with an evanescent optical wave at a planar interface. Opt. Express.

[47] Dahan R (2020). Resonant phase-matching between a light wave and a free-electron wavefunction. Nat. Phys..

[48] Kfir O (2020). Controlling free electrons with optical whispering-gallery modes. Nature.

[49] Kimble HJ (1998). Strong interactions of single atoms and photons in cavity QED. Phys. Scripta.

[50] Park ST, Lin M, Zewail AH (2010). Photon-induced near-field electron microscopy (PINEM): theoretical and experimental. New J. Phys..

[51] Bendaña X, Polman A, García de Abajo FJ (2011). Single-photon generation by electron beams. Nano Lett..

[52] Müller, N. et al. Broadband coupling of fast electrons to high-Q whispering-gallery mode resonators. *ACS Photon*. acsphotonics.1c00456 (2021).

[53] Christopher J (2020). Electron-driven photon sources for correlative electron-photon spectroscopy with electron microscopes. Nanophotonics.

[54] Polman A, Kociak M, García de Abajo FJ (2019). Electron-beam spectroscopy for nanophotonics. Nat. Mater..

[55] Brasch V (2016). Photonic chip– based optical frequency comb using soliton Cherenkov radiation. Science.

[56] Liu J (2021). High-yield, wafer-scale fabrication of ultralow-loss, dispersion-engineered silicon nitride photonic circuits. Nat. Commun..

[57] Pfeiffer MHP (2016). Photonic Damascene process for integrated high-Q microresonator based nonlinear photonics. Optica.

[58] Leedle KJ (2015). Dielectric laser acceleration of sub-100 keV electrons with silicon dual-pillar grating structures. Opt. Lett..

[59] Kippenberg TJ, Spillane SM, Vahala KJ (2002). Modal coupling in traveling-wave resonators. Opt. Lett..

[60] Pomarico E (2018). meV resolution in laser-assisted energy-filtered transmission electron microscopy. ACS Photon..

[61] Malitson IH (1965). Interspecimen comparison of the refractive index of fused silica. J. Opt. Soc. Am..

[62] Brasch V, Chen Q-F, Schiller S, Kippenberg TJ (2014). Radiation hardness of high-Q silicon nitride microresonators for space compatible integrated optics. Opt. Express.

[63] Kippenberg, T. J., Gaeta, A. L., Lipson, M. & Gorodetsky, M. L. Dissipative Kerr solitons in optical microresonators. *Science* **361**, eaan8083 (2018). 10.1126/science.aan8083 30093576

[64] Marin-Palomo P (2017). Microresonator-based solitons for massively parallel coherent optical communications. Nature.

[65] Obrzud E (2019). A microphotonic astrocomb. Nat. Photon..

[66] Suh M-G (2019). Searching for exoplanets using a microresonator astrocomb. Nat. Photon..

[67] Pfeiffer MHP, Liu J, Geiselmann M, Kippenberg TJ (2017). Coupling ideality of integrated planar high-Q microresonators. Phys. Rev. Appl..

[68] Liu J (2018). Double inverse nanotapers for efficient light coupling to integrated photonic devices. Opt. Lett..

[69] Liu J (2016). Frequency-comb-assisted broadband precision spectroscopy with cascaded diode lasers. Opt. Lett..

[70] Feist A (2017). Ultrafast transmission electron microscopy using a laser-driven field emitter: femtosecond resolution with a high coherence electron beam. Ultramicroscopy.

[71] Piazza L (2013). Design and implementation of a fs-resolved transmission electron microscope based on thermionic gun technology. Chem. Phys..

[72] Houdellier F, Caruso G, Weber S, Kociak M, Arbouet A (2018). Development of a high brightness ultrafast transmission electron microscope based on a laser-driven cold field emission source. Ultramicroscopy.

[73] Yurtsever A, Zewail AH (2009). 4D nanoscale diffraction observed by convergent-beam ultrafast electron microscopy. Science.

[74] Feist A, Rubiano da Silva N, Liang W, Ropers C, Schäfer S (2018). Nanoscale diffractive probing of strain dynamics in ultrafast transmission electron microscopy. Struct. Dynam..

[75] Schliep KB, Quarterman P, Wang J-P, Flannigan DJ (2017). Picosecond Fresnel transmission electron microscopy. Appl. Phys. Lett..

[76] Rubiano da Silva N (2018). Nanoscale mapping of ultrafast magnetization dynamics with femtosecond Lorentz microscopy. Phys. Rev. X.

[77] Cao G, Jiang S, Åkerman J, Weissenrieder J (2021). Femtosecond laser driven precessing magnetic gratings. Nanoscale.

[78] Lagos MJ, Trügler A, Hohenester U, Batson PE (2017). Mapping vibrational surface and bulk modes in a single nanocube. Nature.

[79] Hachtel JA (2019). Identification of site-specific isotopic labels by vibrational spectroscopy in the electron microscope. Science.

[80] Li X (2021). Three-dimensional vectorial imaging of surface phonon polaritons. Science.

[81] Kahl, F. et al. in *Advances in Imaging and Electron Physics* (eds Hawkes, P. & Hytch, M.) Vol. 212, 35–70 (Elsevier Inc., 2019).

