# Supplementary material for: Integrated photonics enables continuous-beam electron phase modulation
Source: Nature. 2021 Dec 22;600(7890):653–8. doi: 10.1038/s41586-021-04197-5 (PMC8695378; doi:10.1038/s41586-021-04197-5)
Supplement: Supplementary file 1 — This Supplementary Information file contains Sections 1–4, including Supplementary Figures 1–3 and additional references. Section 1: Analytical equations used for fitting optical resonance; Section 2: Quantum optical description of electron–photon interaction; Section 3: Estimation of transverse beam detection; Section 4: Integrated photonics platforms for electron beam modulation. [file 41586_2021_4197_MOESM1_ESM.pdf]

nature portfolio

https://doi.org/10.1038/s41586-021-04197-5

Supplementary information

Integrated photonics enables continuous-beam electron phase modulation

In the format provided by the authors and unedited

Supplementary Information for Integrated photonics enables continuous-beam electron phase modulation

Jan-Wilke Henke, Arslan Sajid Raja, Armin Feist, Guanhao Huang, Germaine Arend, Yujia Yang, F. Jasmin Kappert, Rui Ning Wang, Marcel Möller, Jiahe Pan, Junqiu Liu, Ofer Kfir, Claus Ropers, Tobias J. Kippenberg

October 20, 2021

1 Analytical equations used for fitting optical resonance

The surface Rayleigh or bulk scattering in the microresonator leads to the coupling of clockwise (a_{cw}) and counter-clockwise (a_{ccw}) modes [1]. Assuming both modes have degenerate frequencies ω with modal coupling rate γ, the Hamiltonian of the microresonator system reads

$$H = \hbar\omega(a^{\dagger}_{\mathrm{cw}}a_{\mathrm{cw}} + a^{\dagger}_{\mathrm{ccw}}a_{\mathrm{ccw}}) + \hbar\gamma(a_{\mathrm{cw}} + a^{\dagger}_{\mathrm{cw}})(a_{\mathrm{ccw}} + a^{\dagger}_{\mathrm{ccw}}). \tag{1}$$

Considering the laser's frame with frequency $\omega_l = \omega + \Delta$, under the rotating wave approximation, the Hamiltonian reads

$$H = -\hbar\Delta(a^{\dagger}_{\mathrm{cw}}a_{\mathrm{cw}} + a^{\dagger}_{\mathrm{ccw}}a_{\mathrm{ccw}}) + \hbar\gamma(a_{\mathrm{cw}}a^{\dagger}_{\mathrm{ccw}} + a^{\dagger}_{\mathrm{cw}}a_{\mathrm{ccw}}). \tag{2}$$

After considering coupling to the bus waveguide with rate κ_{ex} and losses to the environment with rate κ_0, this results in the Langevin equation (ignoring vacuum fluctuation)

$$\dot{a}_{\mathrm{cw}} = (-\kappa/2 + i\Delta)a_{\mathrm{cw}} - i\gamma a_{\mathrm{ccw}} + \sqrt{\eta\kappa}a_{\mathrm{cw,in}} \tag{3}$$

$$\dot{a}_{\mathrm{ccw}} = (-\kappa/2 + i\Delta)a_{\mathrm{ccw}} - i\gamma a_{\mathrm{cw}}, \tag{4}$$

where $\kappa = \kappa_{\mathrm{ex}} + \kappa_0$ describes the total loss rate and $\eta = \kappa_{\mathrm{ex}}/\kappa$ denotes the coupling efficiency. The stationary solution of the intracavity fields can be easily obtained as

$$a_{\mathrm{cw}} = \frac{-\sqrt{\eta\kappa}a_{\mathrm{cw,in}}}{-\kappa/2 + i\Delta + \frac{\gamma^2}{-\kappa/2+i\Delta}} \tag{5}$$

$$a_{\mathrm{ccw}} = \frac{-i\gamma a_{\mathrm{cw}}}{-\kappa/2 + i\Delta}. \tag{6}$$

The cavity transmission, reflection and dissipation are then obtained from the input-output formalism $\mathcal{O}_{\mathrm{out}} = \mathcal{O}_{\mathrm{in}} - \sqrt{\kappa_{\mathrm{ex}}}\mathcal{O}$,

$$P_{\mathrm{t}}/\hbar\omega = |a_{\mathrm{cw,out}}|^2 = |a_{\mathrm{cw,in}} - \sqrt{\eta\kappa}a_{\mathrm{cw}}|^2 \tag{7}$$

$$P_{\mathrm{r}}/\hbar\omega = |a_{\mathrm{ccw,out}}|^2 = |-\sqrt{\eta\kappa}a_{\mathrm{ccw}}|^2 \tag{8}$$

$$P_{\mathrm{diss}}/\hbar\omega = |-\sqrt{(1-\eta)\kappa}a_{\mathrm{cw}}|^2 + |-\sqrt{(1-\eta)\kappa}a_{\mathrm{ccw}}|^2. \tag{9}$$

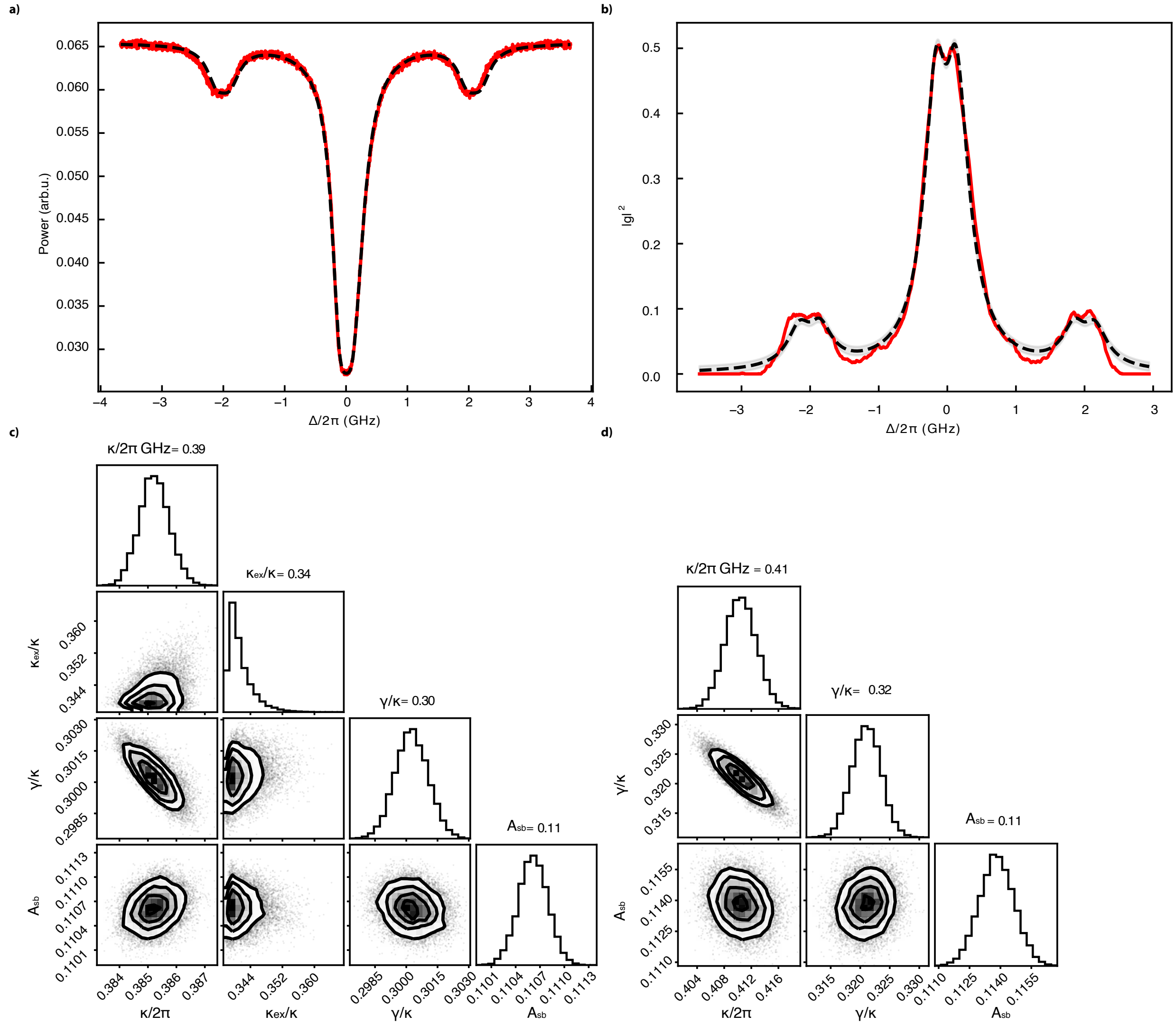

Figure 1: **a)** An optical transmission scan measured at the output of the chip is fitted using Eq.(7). The sidebands generated via an electro-optic modulator are used to calibrate the frequency. **b)** Fitting to the fitted g frequency sweep using Eq.(10). Note that the frequency sweep line shape difference near the resonance is due to the coupling to the frequency degenerate counter-clockwise optical mode. **c,d)** The Markov chain Monte Carlo random walk corner plot of the fitting to the optical data and the fitted g data. The fitted system key parameters (cavity decay rate κ, splitting ratio γ/κ, sideband ratio A_{sb}) of the two frequency sweeps are all within 7% discrepancy, indicating great consistency between the optical and electron spectroscopic measurements. Also strong correlation between the fitted γ and κ is observed for both fittings, indicating the necessity of applying the coupled modes model to correctly extract the cavity decay rate.

And the intracavity photon numbers are simply

$$n_{\mathrm{cw}} = \left|\frac{-\sqrt{\eta\kappa}}{-\kappa/2+i\Delta+\frac{\gamma^2}{-\kappa/2+i\Delta}}\right|^2 \dot{n}_{\mathrm{cw,in}} \tag{10}$$

$$n_{\mathrm{ccw}} = \left|\frac{-i\gamma}{-\kappa/2+i\Delta}\right|^2 n_{\mathrm{cw}}. \tag{11}$$

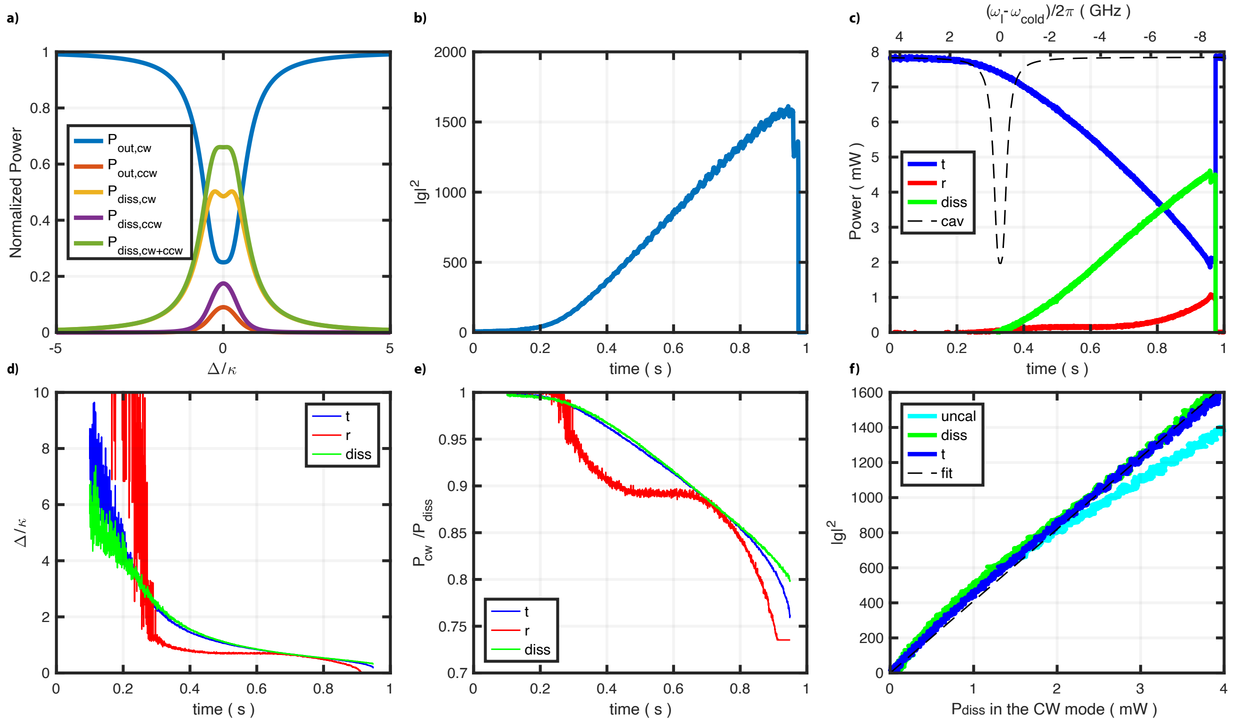

Figure 2: **a)** Simulated power distribution in the optical system when pump is along clockwise direction, generated using parameters fitted from the frequency sweep measurement shown in Fig.1. **b)** $|\mathrm{g}|^2$ scan where each point is obtained after fitting the electron energy distribution. **c)** Experimentally measured optical signal of the cavity transmission (blue), reflection (red), and dissipation (green). The resonance shaped (black dashed) curve shows the inferred cold cavity transmission without thermal absorption and Kerr nonlinearity induced cavity frequency shift, which is present in the triangular shaped (blue) curve due to high input power ($\sim > 50\mu\mathrm{W}$). **d)** Calibrated detuning $\Delta(t)$ based on the experimentally measured optical signal in (c). **e)** Calibrated correction factor $P_{\mathrm{diss,cw}}/P_{\mathrm{diss}}$ from the detuning plot (d). The reflection calibration is empirically erroneous. This effect can be attributed to the etalon formed by the chip facets, by which the transmission signal is less affected. **f)** The calibrated dissipated power in the clockwise mode shows linear relation with $|g|^2$ from different measurement channels (transmission and dissipation).

Eq.(7) and (10) are used for fitting the frequency sweep of the optical transmission $P_t(\Delta)$. The frequency sweep fitting was done using the Markov chain Monte Carlo (MCMC) methods [2], with the optical sidebands $\pm\Omega_{\mathrm{sb}}$ and absorption induced cavity frequency shift $\chi_{\mathrm{th}}P_{\mathrm{diss}}(\Delta)$ included in the models. The fitting function is $F_{\mathrm{fit}}(\Delta) = \sum_{n=-1,0,1} F_i(\Delta + n\Omega_{\mathrm{sb}} + \chi_{\mathrm{th}}P_{\mathrm{diss}}(\Delta))$, where F_i is either P_t for optical measurement or n_a for measurement of the electron-light coupling g. The fitting results are shown in Fig.1, and the fitted system parameters show great consistency between the two distinct measurements.

For the power sweep calibration, Eq.(7), (10) and (11) are used for calibrating the clockwise dissipated power $\tilde{P}_{\mathrm{diss},cw}(t)$. The detuning $\tilde{\Delta}(t)$ was extracted from the experimentally measured $\tilde{P}_{\mathrm{t}}(t)$ (Fig.2(b), suffers the least from background noise) using the fitted resonator parameters (κ, η, γ) from the frequency sweep (Fig.2(a)(c)). Then the clockwise dissipated power $\tilde{P}_{\mathrm{diss},cw}(t)$ (Fig.2(e)) was calibrated from the experimentally measured transmission power $\tilde{P}_{\mathrm{t}}(t)$ and the calibrated $\tilde{\Delta}(t)$ by $\tilde{P}_{\mathrm{diss},cw}(t) = \frac{P_{\mathrm{diss},cw}(\Delta(t))}{P_{\mathrm{t}}(\Delta(t))}\tilde{P}_{\mathrm{t}}(t)$. We later calculate the characteristic coupled optical power $P = n_{\mathrm{cw}}\hbar\omega\kappa$ by scaling the dissipated power $P = \frac{\kappa}{\kappa_0} \times \tilde{P}_{\mathrm{diss},cw}$, and plot it against the fitted coupling constant $|g(t)|^2$ (fig. 2f), one could find the linear

relation as is expected in theory. The observable oscillations in the linear dependence of $|g|^2$ on the clockwise dissipated power are related to a 50 Hz noise in the beam position leading to variations in electron-light coupling strength. To eliminate this 50 Hz noise, we binned the retrieved coupling strength in time intervals of 20 ms. The resulting power dependence is shown in main text figure 2.e).

2 Quantum optical description of electron-photon interaction

As discussed in the main manuscript, for electron-photon interaction, it is more natural to work in the velocity gauge [3]. For simplicity, we reduce the problem to one dimension ($\hat{z}$) along the electron propagation direction. The vector potential is quantized as [4]

$$\hat{A} = \hat{A}(z) = \sqrt{\frac{\hbar}{2\epsilon\omega V}}(u(z)a + u^*(z)a^\dagger),$$

where ϵ is the optical permittivity, ω is the optical frequency, V is the effective optical mode volume, and $u(z)$ is the z projection (along the propagation direction of electron $\vec{k}_e$) of the vector mode function $\vec{u}(\vec{r})$ which satisfies

$$\int_V |\vec{u}(\vec{r})|^2 d\vec{r}^3 = V.$$

We choose the electron plane wave states as the basis,

$$|k_e\rangle = \int dz|z\rangle\langle z||k_e\rangle = \lim_{L\to\infty}\int_{-L/2}^{L/2} dz L^{-1/2}\exp\left(ik_e z\right)|z\rangle$$

$$\mathbf{1} = \sum_{k_e}|k_e\rangle\langle k_e| = \lim_{L\to\infty}\frac{L}{2\pi}\int dk_e|k_e\rangle\langle k_e|.$$

The coupling term can then be readily calculated as

$$\begin{aligned}
H_1 &= \frac{e}{2m}(\hat{p}\hat{A} + \hat{A}\hat{p}) \\
&= \sqrt{\frac{\hbar}{2\epsilon\omega V}}\frac{e}{2m}\sum_{k_e}|k_e\rangle\langle k_e|\cdot\hat{p}\cdot\int_{L_c} dz|z\rangle\langle z|(u(z)a + u^*(z)a^\dagger)\sum_{k_e'}|k_e'\rangle\langle k_e'| + h.c. \\
&= \lim_{L\to\infty}\sqrt{\frac{\hbar}{2\epsilon\omega V}}\frac{e}{mL}\left(\sum_{\Delta k_e}\sum_{k_e}\hbar(k_e - \Delta k_e/2)\int_{L_c} dz e^{-i\Delta k_e\cdot z}u(z)a|k_e\rangle\langle k_e - \Delta k_e| + \text{h.c.}\right) \\
&= \sqrt{\frac{\hbar e^2 v_e^2}{2\epsilon\omega V}}\left(a\sum_{k_e}\int_{\Delta k_e} d\Delta k_e\frac{k_e - \Delta k_e/2}{k_e^0}\frac{L_{\text{eff}}(\Delta k_e)}{2\pi}|k_e\rangle\langle k_e - \Delta k_e| + \text{h.c.}\right) \\
&= \hbar g_0 a b^\dagger + \hbar g_0^* a^\dagger b,
\end{aligned}$$

with the vacuum coupling rate $g_0 = \sqrt{\frac{e^2 v_e^2}{2\epsilon\hbar\omega V}}$ in which v_e is the electron velocity and e is the electron charge, and the electron transition operator $b = \sum_{k_e}\int_{\Delta k_e} d\Delta k_e \frac{k_e - \Delta k_e/2}{k_e^0}\frac{L^*_{\text{eff}}(\Delta k_e)}{2\pi}|k_e - \Delta k_e\rangle\langle k_e|$. Here, $L_{\text{eff}}(\Delta k_e) = \int_{L_c} dz e^{-i\Delta k_e\cdot z}u(z)$ is the effective interaction length, and represents the phase matching condition between the optical field profile function $u(z)$ along the electron propagation trajectory and the electron wavevector change $e^{-i\Delta k_e\cdot z}$ upon the absorption/emission of a photon. Under conditions of phase matching, the expression can be reduced to the order of the physical length over which the optical field and the electron interact.

In the interaction picture, the scattering matrix, derived from the interaction Hamiltonian, is

$$\begin{aligned}
H_{\text{int}} &= \hbar g_0 a \sum_{k_e} \int_{\Delta k_e} d\Delta k_e \frac{k_e - \Delta k_e/2}{k_e^0} \frac{L_{\text{eff}}(\Delta k_e)}{2\pi} |k_e\rangle\langle k_e - \Delta k_e| e^{-i\omega t} e^{i[E(k_e)-E(k_e-\Delta k_e)]t/\hbar} + \text{h.c.} \\
S &= T \exp\left(-\frac{i}{\hbar}\int_{\tau\to\infty} H_{\text{int}} dt\right) \\
&\approx \exp\left(-i g_0 a \sum_{k_e} \iint_{\tau,\Delta k_e} d\Delta k_e \frac{k_e - \Delta k_e/2}{k_e^0} \frac{L_{\text{eff}}(\Delta k_e)}{2\pi} |k_e\rangle\langle k_e - \Delta k_e| e^{i([E(k_e)-E(k_e-\Delta k_e)]/\hbar-\omega)t} dt - \text{h.c.}\right) \\
&= \exp\left(-i g_0 a \sum_{k_e} \int_{\Delta k_e} d\Delta k_e \frac{k_e - \Delta k_e/2}{k_e^0} L_{\text{eff}}(\Delta k_e) |k_e\rangle\langle k_e - \Delta k_e| \delta\left[\frac{E(k_e) - E(k_e - \Delta k_e)}{\hbar} - \omega\right] - \text{h.c.}\right) \\
&= \exp\left(-i g_0 a \sum_{E_e} \frac{k_e - \Delta k/2}{k_e^0} \frac{L_{\text{eff}}(\Delta k)}{\partial_{p_e} E} |E_e\rangle\langle E_e - \hbar\omega| - \text{h.c.}\right) \\
&\approx \exp\left(-i g_0 \frac{L_{\text{eff}}}{v_e} a \sum_{E_e \in [E_e^0 - \epsilon, E_e^0 + \epsilon]} |E_e\rangle\langle E_e - \hbar\omega| - \text{h.c.}\right) \\
&= \exp\left(-i \tilde{g}_0 \tau_{\text{int}} a \tilde{b}^\dagger - \text{h.c.}\right),
\end{aligned}$$

with a re-defined expression of $\tilde{b} = \sum_{E_e \in [E_e^0 - \epsilon, E_e^0 + \epsilon]} |E_e\rangle\langle E_e - \hbar\omega|$. Moreover, $\Delta k \approx \frac{\omega}{v_e}$, defined by $E(k_e) - E(k_e - \Delta k) = \hbar\omega$, is the electron wave-vector change due to the absorption/emission of a photon. The vacuum coupling rate is also re-defined as $\tilde{g}_0 = \eta\sqrt{\frac{e^2 v_e^2}{2\epsilon\hbar\omega V}}$, so that the interaction time $\tau_{\text{int}} = \frac{L_{\text{int}}}{v_e}$ has a very clear physical meaning, which is the fly-by time of the electron over the interaction region L_{int}. Here the phase matching coefficient $\eta = \int dz e^{-i\Delta k \cdot z} u(z)/L_{\text{int}}$ is re-introduced in the vacuum coupling rate $\tilde{g}_0$.

In the presence of a strong coherent laser drive $|\alpha\rangle$, the scattering matrix reduces to a displacement operator on the electron state $S \approx \exp\left(-i g_0 \tau_{\text{int}} \alpha b^\dagger - \text{h.c.}\right)$, which results in the probability distribution on the N_{th} sideband

$$\begin{aligned}
P_N &= |\langle E_e^0 + N\Delta | S | E_e^0\rangle|^2 = J_N(2g)^2 \\
g &= |\alpha g_0 \tau_{\text{int}}| = \sqrt{\frac{n_{\text{ph}} e^2}{2\epsilon\hbar\omega V}} \left|\int dz e^{-i\Delta\cdot z} u(z)\right| \\
&= \frac{e}{2\hbar\omega}\left|\int dz e^{-i\Delta\cdot z} \tilde{E}(z)\right|,
\end{aligned}$$

where the complex field $\tilde{E}(z)$ is related to the physical electric field $E(z,t)$ via $E(z,t) = \text{Real}[\tilde{E}(z)e^{-i\omega t}]$.

3 Estimation of transverse beam deflection

Since inelastic electron-light scattering is typically accompanied by a three-dimensional momentum transfer, we here estimate the transverse deflection of the electron beam upon passing by the resonator structure. For an electron energy of 120 keV, a photon energy of about 0.8 eV (corresponding to a wavelength of 1550 nm) and assuming $E_\varphi = E_z$ (i.e. electron-photon scattering above a straight waveguide), a single photon scattering would lead to a deflection of $\sim 1.5\,\mu$rad. Accordingly, in the case of multi-photon exchange with $N = 250$, as shown in Fig. 2d of the main text, a deflection of < 0.4 mrad is expected. For the measurements presented here, the STEM camera length was chosen such that it enables full beam transmission through the angle-limiting aperture into the spectrometer.

4 Integrated photonics platforms for electron beam modulation

Various integrated photonics platforms have been used for controlling manifold quantum systems [5–15]. The current Si_3N_4-based electron beam phase modulation demonstration could be potentially extended to other well-established photonic integrated platforms showing the broader applicability. Finite element method based simulations are performed to calculate the n_{eff} of the microresonators with different materials by sweeping the frequency and changing the waveguide height. The width of the waveguide is kept at 1.5 μm for all materials (Fig. 3). Different shaded regions are calculated by performing simulations at two different waveguide heights (e.g., solid light-red line GaP: 1500 x 800 nm^2, dashed light-red line GaP: 1500 x 400 nm^2). It is possible to achieve phase matching for electron kinetic energies ranging from 30 to 180 keV by using different established integrated photonic platforms (AlGaAs [16, 17], GaP [18], 4H-SiC [19], Diamond [20], $LiNbO_3$ [21], Si_3N_4 [22], Hydex [22, 23]). Some other prominent platforms, such as Si [24], AlN [25], Ta_2O_5 [26], and SiO_2 [27] are not shown due to overlapping phase matching, but could also be considered for implementing the electron beam phase modulation. In addition to phase matching, other important parameters that also need to be considered are linear propagation loss, light coupling into the chip and optical power handling.

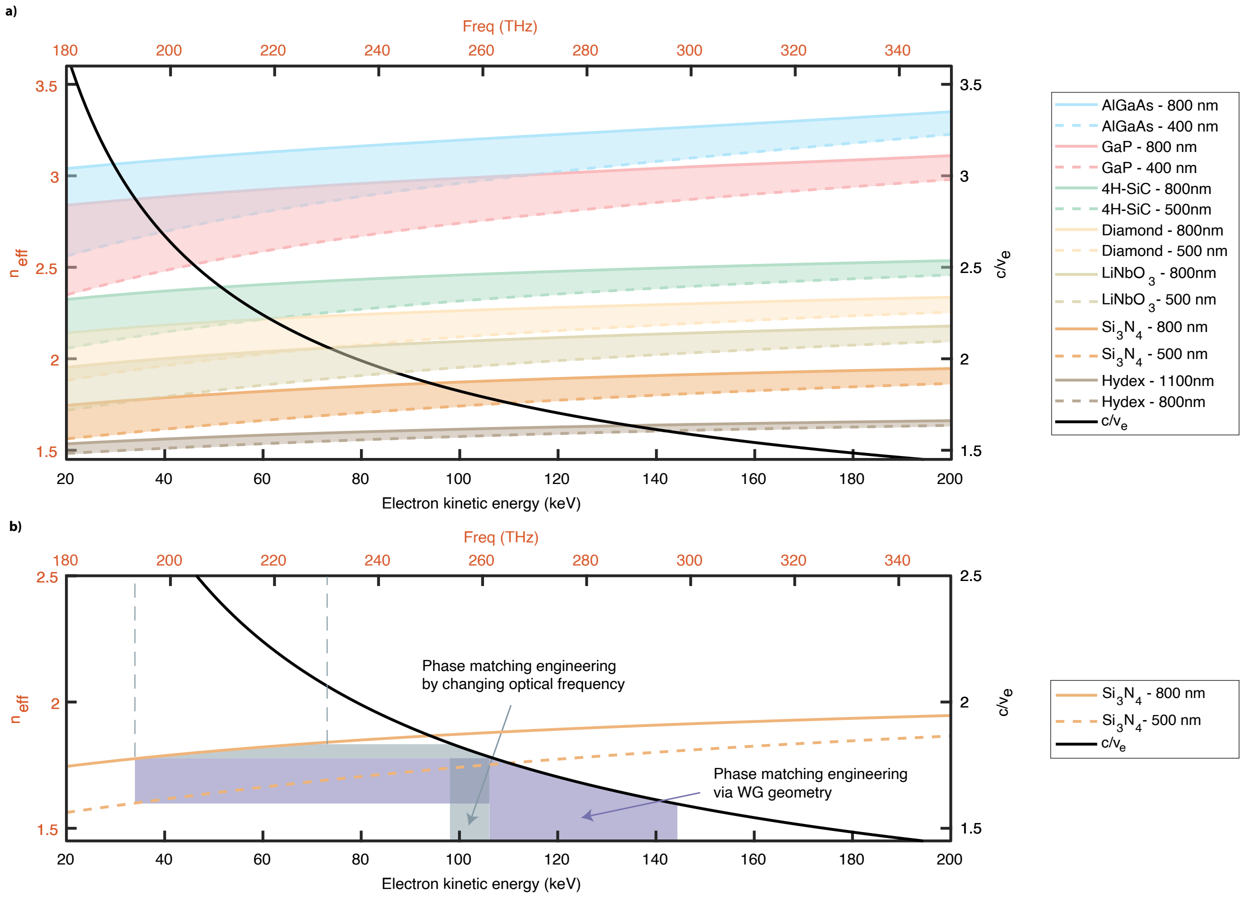

Figure 3: Phase matching condition for different materials and optical frequencies. **a)** For each electron, the electron velocity determines a phase-matched refractive index value, which is accessible through a wide range of optical frequencies and material platforms by engineering of the waveguide geometry. The possible simulated regions of the microresonators' effective index (quasi-TM mode) with 50 μm radius for different materials are achieved by varying either the waveguide dimensions (height) or the optical frequency. Each shaded region shows a possible operating regime for a single integrated platform. Si_3N_4 could provide phase matching from 95 to 145 keV, while phase matching below 80 keV could be implemented using $4H-SiC$ or $LiNbO_3$. b) The two shaded regions show phase matching windows for geometry or frequency tuning. The light purple region can be reached by changing the Si3N4 waveguide height from 800 nm to 500 nm while operating at a single frequency (193 THz). The gray-shaded region can be reached by changing the optical frequency from ~193 THz to 230 THz.

References

[1] Michael L. Gorodetsky, Andrew D. Pryamikov, and Vladimir S. Ilchenko. Rayleigh scattering in high-q microspheres. *J. Opt. Soc. Am. B*, 17(6):1051–1057, Jun 2000.

[2] D. Foreman-Mackey, D. W. Hogg, D. Lang, and J. Goodman. emcee: The mcmc hammer. *PASP*, 125:306–312, 2013.

[3] Armin Feist, Katharina E. Echternkamp, Jakob Schauss, Sergey V. Yalunin, Sascha Schäfer, and Claus Ropers. Quantum coherent optical phase modulation in an ultrafast transmission electron microscope.

Nature, 521(7551):200–203, 2015.

[4] Marlan O Scully and M Suhail Zubairy. Quantum optics, 1999.

[5] J. D. Thompson, T. G. Tiecke, N. P. de Leon, J. Feist, A. V. Akimov, M. Gullans, A. S. Zibrov, V. Vuletić, and M. D. Lukin. Coupling a Single Trapped Atom to a Nanoscale Optical Cavity. *Science*, 340(6137):1202–1205, 2013.

[6] D. E. Chang, J. S. Douglas, A. González-Tudela, C.-L. Hung, and H. J. Kimble. Colloquium: Quantum matter built from nanoscopic lattices of atoms and photons. *Rev. Mod. Phys.*, 90(3):031002, 2018.

[7] Neil V. Corzo, Jérémy Raskop, Aveek Chandra, Alexandra S. Sheremet, Baptiste Gouraud, and Julien Laurat. Waveguide-coupled single collective excitation of atomic arrays. *Nature*, 566(7744):359–362, 2019.

[8] Karan K. Mehta, Colin D. Bruzewicz, Robert McConnell, Rajeev J. Ram, Jeremy M. Sage, and John Chiaverini. Integrated optical addressing of an ion qubit. *Nat. Nanotechnol.*, 11(12):1066–1070, 2016.

[9] Karan K. Mehta, Chi Zhang, Maciej Malinowski, Thanh-Long Nguyen, Martin Stadler, and Jonathan P. Home. Integrated optical multi-ion quantum logic. *Nature*, 586(7830):533–537, 2020.

[10] R. J. Niffenegger, J. Stuart, C. Sorace-Agaskar, D. Kharas, S. Bramhavar, C. D. Bruzewicz, W. Loh, R. T. Maxson, R. McConnell, D. Reens, G. N. West, J. M. Sage, and J. Chiaverini. Integrated multi-wavelength control of an ion qubit. *Nature*, 586(7830):538–542, 2020.

[11] Immo Söllner, Sahand Mahmoodian, Sofie Lindskov Hansen, Leonardo Midolo, Alisa Javadi, Gabija Kiršanskė, Tommaso Pregnolato, Haitham El-Ella, Eun Hye Lee, Jin Dong Song, Søren Stobbe, and Peter Lodahl. Deterministic photon–emitter coupling in chiral photonic circuits. *Nat. Nanotechnol.*, 10(9):775–778, 2015.

[12] Peter Lodahl, Sahand Mahmoodian, and Søren Stobbe. Interfacing single photons and single quantum dots with photonic nanostructures. *Rev. Mod. Phys.*, 87(2):347–400, 2015.

[13] Feng Liu, Alistair J. Brash, John O'Hara, Luis M. P. P. Martins, Catherine L. Phillips, Rikki J. Coles, Benjamin Royall, Edmund Clarke, Christopher Bentham, Nikola Prtljaga, Igor E. Itskevich, Luke R. Wilson, Maurice S. Skolnick, and A. Mark Fox. High Purcell factor generation of indistinguishable on-chip single photons. *Nature Nanotech*, 13(9):835–840, 2018.

[14] Justin C. Norman, Daehwan Jung, Yating Wan, and John E. Bowers. Perspective: The future of quantum dot photonic integrated circuits. *APL Photonics*, 3(3):030901, 2018.

[15] A. Sipahigil, R. E. Evans, D. D. Sukachev, M. J. Burek, J. Borregaard, M. K. Bhaskar, C. T. Nguyen, J. L. Pacheco, H. A. Atikian, C. Meuwly, R. M. Camacho, F. Jelezko, E. Bielejec, H. Park, M. Lončar, and M. D. Lukin. An integrated diamond nanophotonics platform for quantum-optical networks. *Science*, 354(6314):847–850, 2016.

[16] Minhao Pu, Luisa Ottaviano, Elizaveta Semenova, and Kresten Yvind. Efficient frequency comb generation in algaas-on-insulator. *Optica*, 3(8):823–826, Aug 2016.

[17] Lin Chang, Weiqiang Xie, Haowen Shu, Qi-Fan Yang, Boqiang Shen, Andreas Boes, Jon D Peters, Warren Jin, Chao Xiang, Songtao Liu, et al. Ultra-efficient frequency comb generation in algaas-on-insulator microresonators. *Nature communications*, 11(1):1–8, 2020.

[18] Dalziel J Wilson, Katharina Schneider, Simon Hönl, Miles Anderson, Yannick Baumgartner, Lukas Czornomaz, Tobias J Kippenberg, and Paul Seidler. Integrated gallium phosphide nonlinear photonics. *Nature Photonics*, 14(1):57–62, 2020.

[19] Daniil M Lukin, Constantin Dory, Melissa A Guidry, Ki Youl Yang, Sattwik Deb Mishra, Rahul Trivedi, Marina Radulaski, Shuo Sun, Dries Vercruysse, Geun Ho Ahn, et al. 4h-silicon-carbide-on-insulator for integrated quantum and nonlinear photonics. *Nature Photonics*, 14(5):330–334, 2020.

[20] BJM Hausmann, I Bulu, V Venkataraman, P Deotare, and Marko Lončar. Diamond nonlinear photonics. *Nature Photonics*, 8(5):369–374, 2014.

[21] Mian Zhang, Cheng Wang, Rebecca Cheng, Amirhassan Shams-Ansari, and Marko Lončar. Monolithic ultra-high-q lithium niobate microring resonator. *Optica*, 4(12):1536–1537, Dec 2017.

[22] David J Moss, Roberto Morandotti, Alexander L Gaeta, and Michal Lipson. New cmos-compatible platforms based on silicon nitride and hydex for nonlinear optics. *Nature photonics*, 7(8):597–607, 2013.

[23] Luca Razzari, David Duchesne, Marcello Ferrera, Roberto Morandotti, S Chu, BE Little, and DJ Moss. Cmos-compatible integrated optical hyper-parametric oscillator. *Nature Photonics*, 4(1):41–45, 2010.

[24] Mohammad Soltani, Siva Yegnanarayanan, Qing Li, and Ali Adibi. Systematic engineering of waveguide-resonator coupling for silicon microring/microdisk/racetrack resonators: theory and experiment. *IEEE Journal of Quantum Electronics*, 46(8):1158–1169, 2010.

[25] Hojoong Jung, Chi Xiong, King Y Fong, Xufeng Zhang, and Hong X Tang. Optical frequency comb generation from aluminum nitride microring resonator. *Optics letters*, 38(15):2810–2813, 2013.

[26] Hojoong Jung, Su-Peng Yu, David R Carlson, Tara E Drake, Travis C Briles, and Scott B Papp. Tantala kerr nonlinear integrated photonics. *Optica*, 8(6):811–817, 2021.

[27] Ki Youl Yang, Dong Yoon Oh, Seung Hoon Lee, Qi-Fan Yang, Xu Yi, Boqiang Shen, Heming Wang, and Kerry Vahala. Bridging ultrahigh-q devices and photonic circuits. *Nature Photonics*, 12(5):297–302, 2018.
